# On-Top Plasty at the Level of the Metatarsal Neck for Treatment of Polydactyly

**DOI:** 10.3389/fped.2022.931148

**Published:** 2022-06-27

**Authors:** Wei Liao, Li Jiang, Pengfei Zheng

**Affiliations:** Department of Orthopaedic Surgery, Children's Hospital of Nanjing Medical University, Nanjing, China

**Keywords:** polydactyly, foot, on-top plasty, metatarsals, wide forefoot

## Abstract

Polydactyly is a common deformity of the limbs, and excision of the extra digit has shown good results in the vast majority of patients. However, this treatment approach may not suitable for all cases of polydactyly. Some complex surgical procedures are required to treat rare forms of polydactyly and achieve satisfactory correction. Here, we report the use of on-top plasty technique for treating polydactyly between the 4th and 5th metatarsals with concomitant angulation of the 5th metatarsophalangeal joint. We performed the first osteotomy at the neck of metatarsal bone by “grafting” the distal polydactyly with the normal axis to the 5th metatarsal bone. Excision of the extra toe was accompanied by simultaneous restoration of the 5th toe axis and decrease in the width of the forefoot. Finally, both appearance and function could be improved. With this novel method, the complete osteoarticular structure and weight-bearing structure of foot were well-reconstructed. Based on the findings, we recommend that for the surgery of polydactyly, the beneficial parts should be preserved for reconstruction, and the tailored and personalized approach could be adopted.

## Introduction

Polydactyly is the most common congenital toe deformity. It has an incidence of 5.1 in every 10,000 individuals in Japan, and postaxial polydactyly accounts for 86% of all cases (1). Polydactyly usually occurs on the fibular side, and this increases the width of the foot and makes it difficult to wear shoes. According to the position of extra digits, Lee et al. divided axial polydactyly into seven categories (2). Most of these seven types of polydactyly can be treated satisfactorily by excising the extra digit. However, there are rare forms of polydactyly that require more complex surgical procedures to achieve satisfactory correction. For example, Satosh et al. (3) reported a case of polydactyly with short toe deformity that was successfully treated with an “on-top plasty” technique involving osteotomy and transfer at the phalangeal level. They were able to improve the length of the toe with this technique. However, in cases with lateral varus deformity, phalange-level osteotomy and transfer do not solve the problem of forefoot width and pain. Therefore, complex cases of polydactyly require complex treatment approaches that are specific and tailored to meet the needs of different cases.

In this study, we described the new method well-suitable for treating the postaxial polydactyly with an unusually wide foot, which was radical metatarsal neck osteotomy and transfer via the on-top plasty technique. Through this method, the extra digit was excised, the toe line axis was corrected, the function of foot arch was restored, and the aesthetic appearance was improved. We hope that our experience will help in treatment decision-making in similar situation in the future.

## Materials and Methods

**Figure 1:**
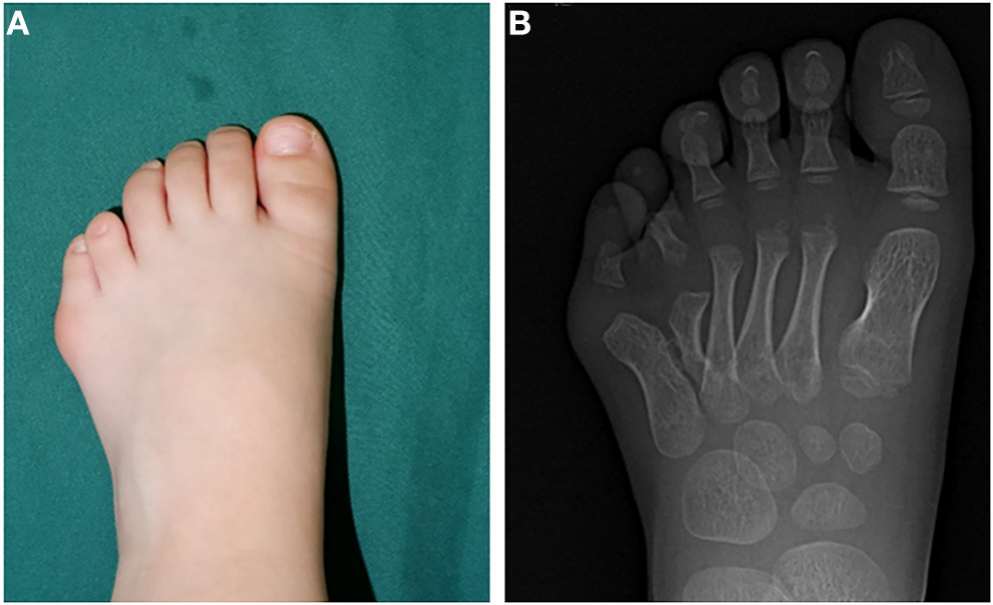
Preoperative morphological and imaging features. **(A)** Preoperative photos depicting the appearance of the affected foot. **(B)** Preoperative radiograph showing malformed fifth metatarsal and wide forefoot.

The patient involved in the study was a 4 years and 3 months old boy. He was admitted because he experienced difficulty while wearing his shoes and pain in the fifth toe. Clinical examination showed postaxial polydactyly with an abnormally enlarged forefoot (Figure 1A). Preoperative radiography findings indicated polydactyly at the site between the 4th and 5th metatarsal, angulation of the metatarsal joint of the fifth toe toward the fibular side, and an unusually wide forefoot (Figure 1B).

**Figure 2:**
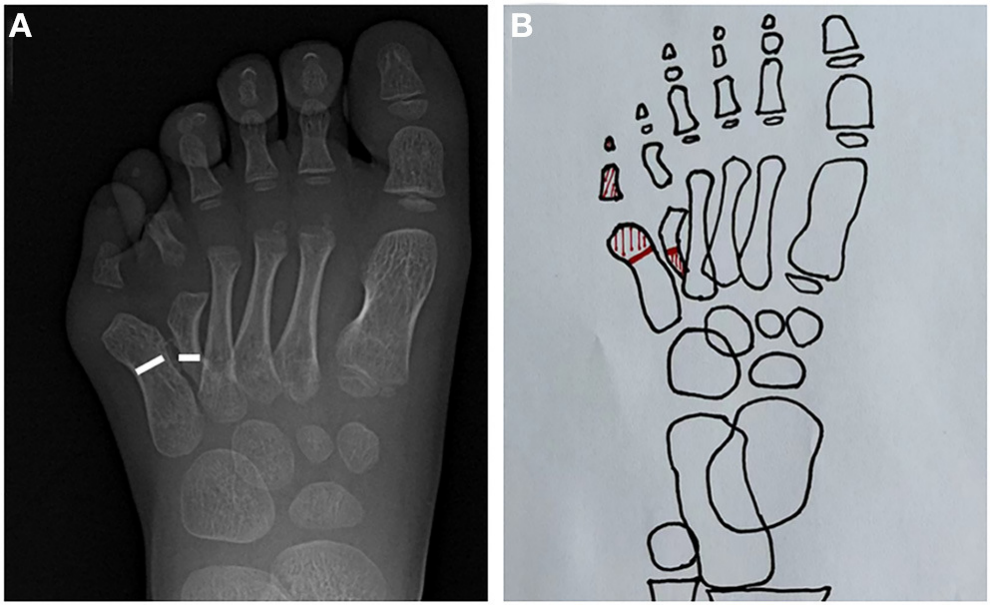
Radiographic and schematic depiction of the surgical process. **(A)** Radiographic image depicting the lateral column and medial column osteotomy line. **(B)** Schematic diagram of the surgical process. The red shaded part represents the excised part.

We used the on-top plasty technique to reconstruct this complex deformity. Indications of this technique were that the medial toe with good appearance and metatarsal dysplasia, and the malformed and distorted lateral toe with complete metatarsal and phalangeal bone and joint structure, requiring combined reconstruction to obtain complete phalange. The following steps were performed during preoperative planning. First, the distal part of the lateral column of malformation, including the metatarsal phalangeal joint, was resected. In the next step, the proximal end of the medial column was resected. In the third step, the distal end of the normal medial row axis was grafted to the lateral row metatarsals to restore the axis and appearance of toe (Figure 2).

For the main procedure, a “Z” skin incision was made in the dorsal skin of foot with the patient under general anesthesia. During the procedure, we observed multiple digits protruding from the space between the 4th and 5th metatarsals. The fifth metatarsal neck was abnormally biased toward the peroneal side, and the metatarsal toe joint was angulated toward the peroneal side. We performed osteotomy at the metatarsal neck of the 5th metatarsal and polydactyly. The selection of osteotomy level was mainly based on whether there was short toe deformity. If there was no short toe deformity, osteotomy should be performed at the same level. In this case, short toe need to be lengthened by metatarsal transfer, so the medial metatarsal osteotomy line should be moved to the proximal end and the lateral metatarsal osteotomy line should be moved to the distal end, accordingly, so as to increase metatarsal length and improve the length of toe. Then, the distal metatarsal bone of the 5th toe and the proximal part of the polydactyly were resected. The vascular and nerve tracts at the distal end of polydactyly were completely preserved. The distal end of polydactyly was aligned with the 5th metatarsal bone and fixed with Kirschner wire. At the osteotomy level, the periosteum was sutured carefully, layer by layer, to the skin. After surgery, a cast was placed and used for 6 weeks. The Kirschner wire was removed at postoperative 6 weeks, and weight-bearing functional exercises were started gradually.

## Results

**Figure 3:**
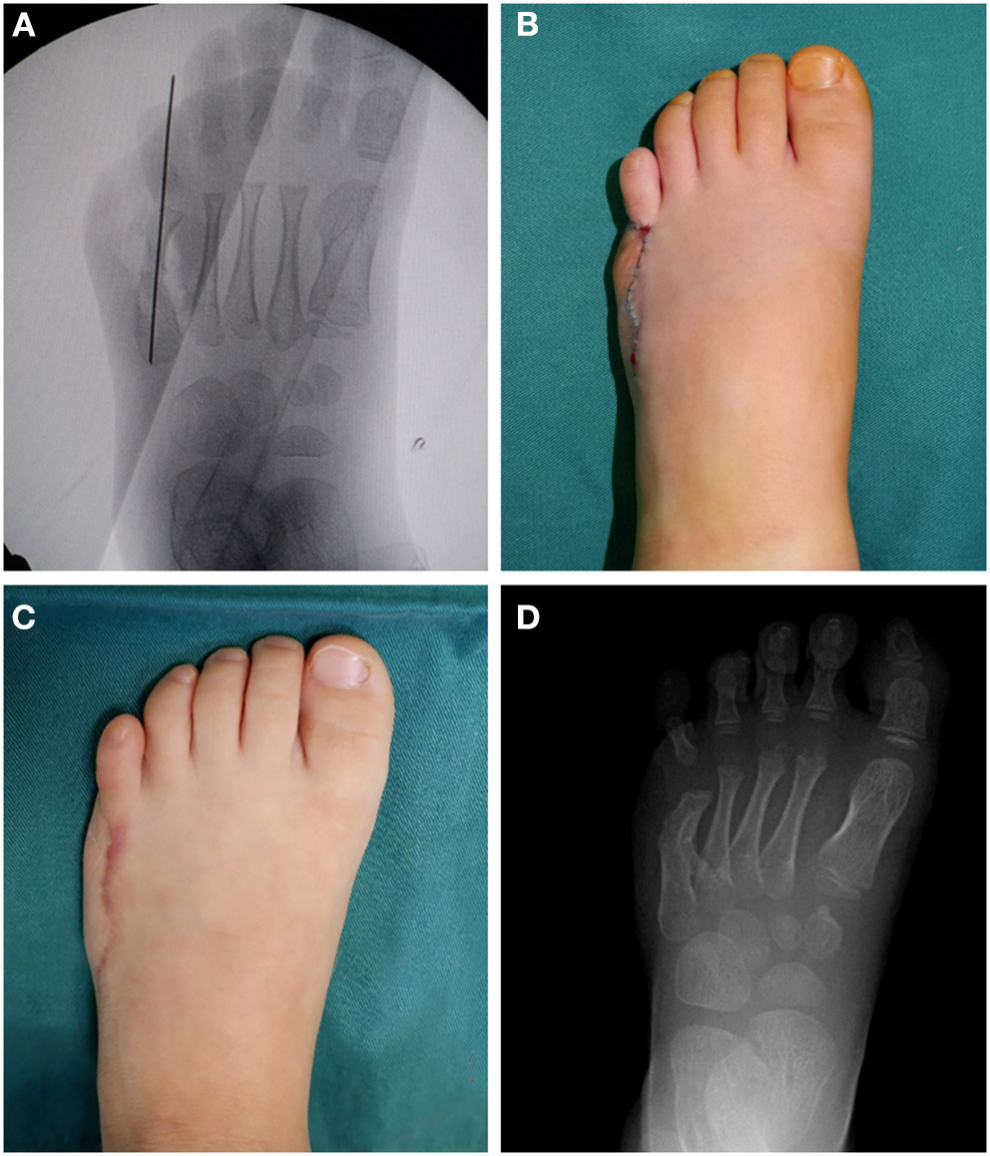
Intraoperative fluoroscopic images, immediate postoperative appearance and 1-year follow-up findings. **(A)** Intraoperative fluoroscopic radiographic images showing the fifth ray axis was restored and the wide forefoot was improved. **(B)** Immediate postoperative photos showing the appearance of the foot. **(C)** Photos showing the appearance of the operated foot at 1-year after surgery. **(D)** Radiograph showing good healing ang the fifth ray axis was restored, taken 1-year after surgery.

**Figure 4:**
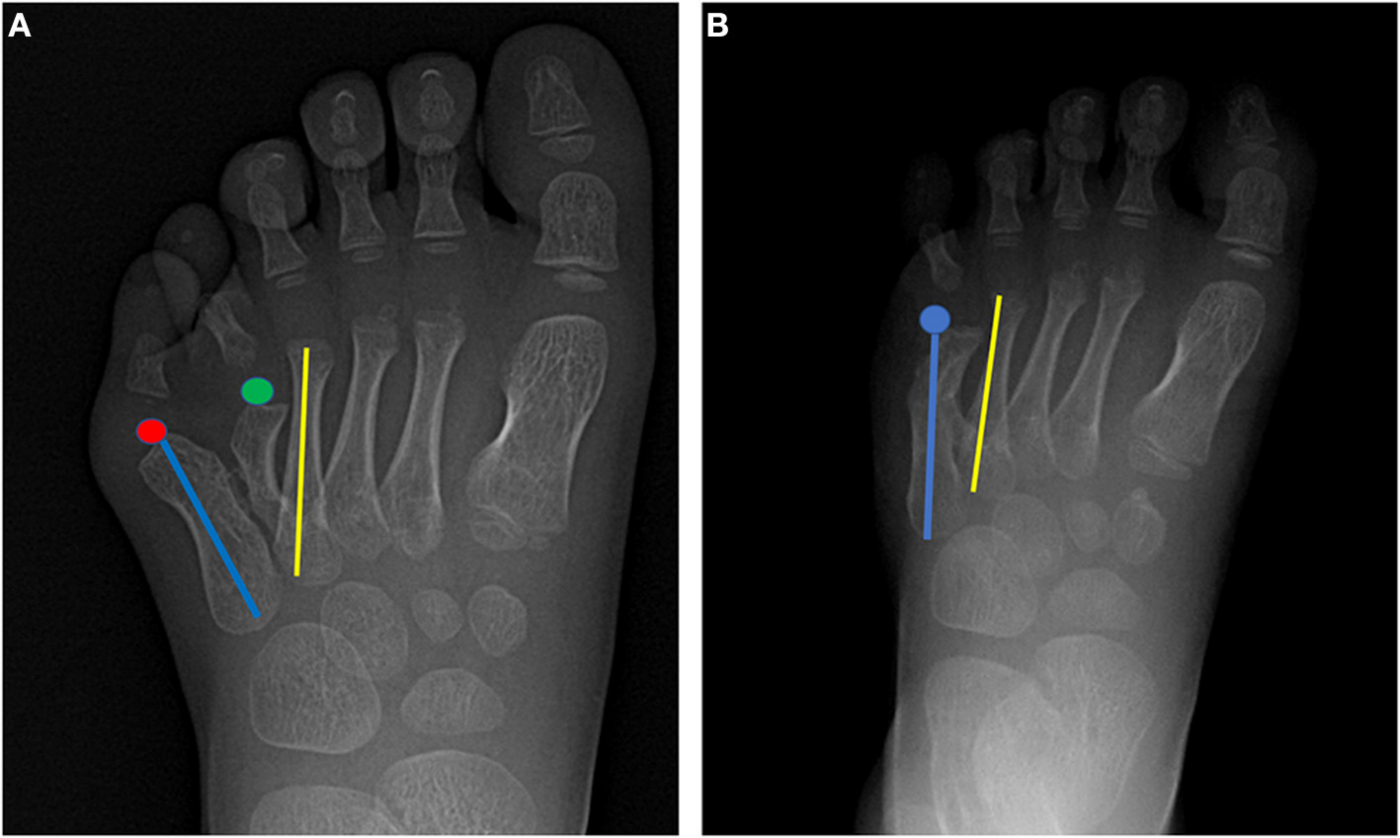
Pre- and postoperative radiographs for comparison of foot morphology. **(A)** The red dots represent the centers of the lateral metatarsal bones, and the green dots represent the centers of the medial metatarsal bones. The blue line represents the axis of the fifth metatarsal, and the yellow line represents the axis of the fourth metatarsal. **(B)** The blue line represents the axis of the fifth metatarsal bone after surgery. The blue dot represents the center of the fifth metatarsal bone after surgery; the sequence of the fifth toe line returned to normal after surgery.

**Figure 5:**
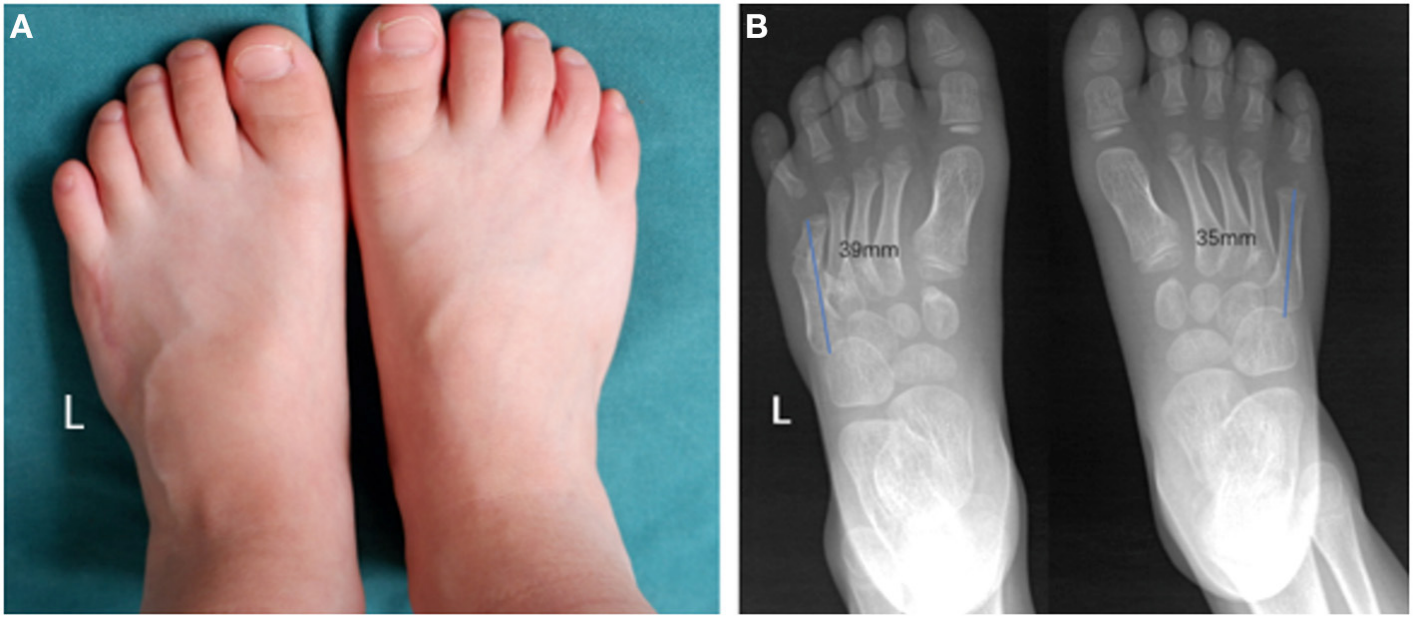
Postoperative morphological and radiologic characteristic of feet. **(A)** The appearance of left foot recovered well 1-year after operation, and the length of the fifth toe was the same as that of the unimpaired side. **(B)** The fifth digit column of left foot was lengthened. The postoperative length of the fifth metatarsal in the left foot was 39 mm and the fifth metatarsal in the right foot was 35 mm.

**Figure 6:**
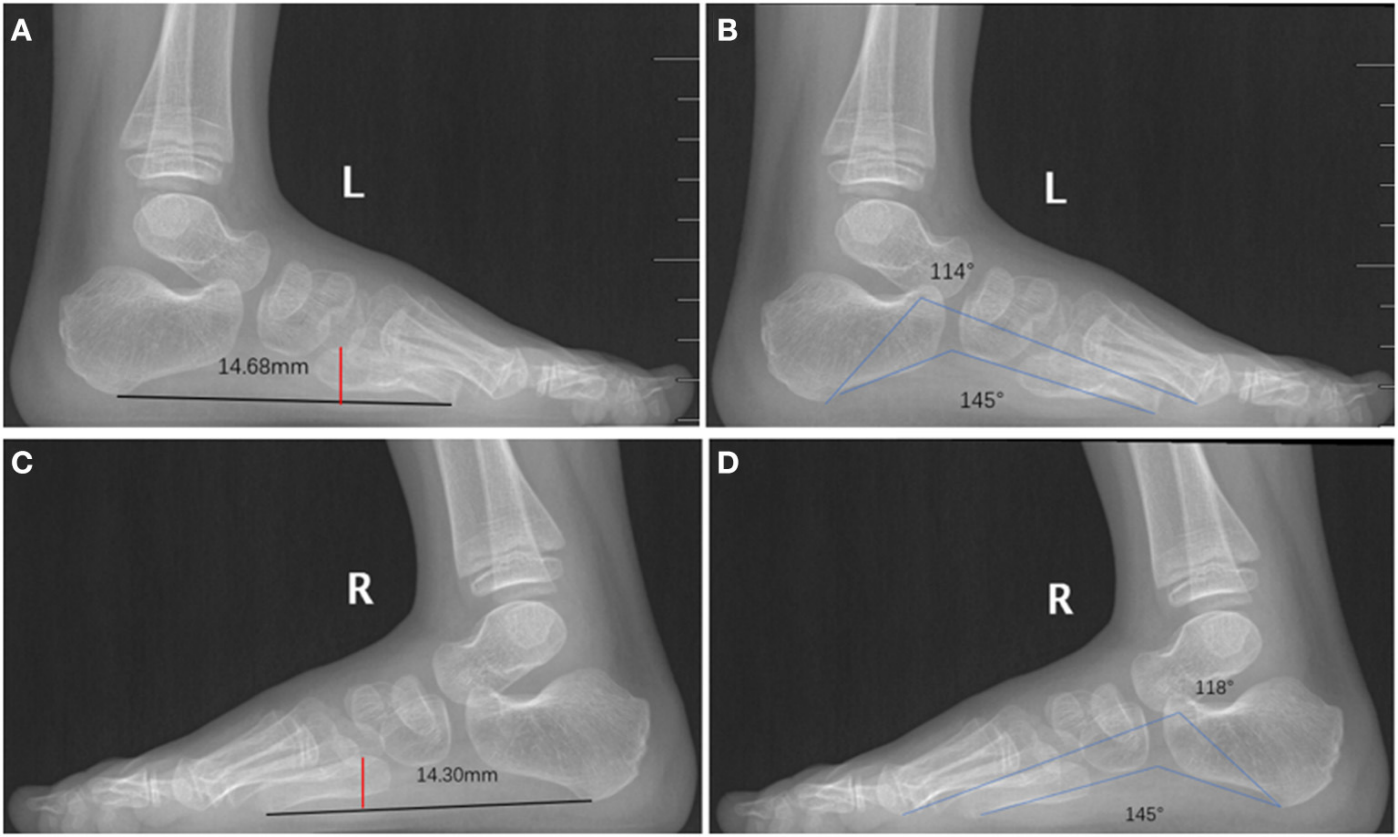
Postoperative medial and lateral arches of feet. **(A)** The height of the fifth metatarsal bone of left foot was 14.68 mm. **(B)** The medial arch of left foot was 114° and the lateral arch was 145°. **(C)** The height of the fifth metatarsal of right foot was 14.30 mm. **(D)** The medial arch of right foot was 118° and the lateral arch was 145°. The reconstructed lateral arch is similar to the unimpaired side.

Intraoperative radiographs showed that on top-plasty of the metatarsal neck level and 5th digit sequence was restored (Figure 3A). Immediately after surgery, the appearance was improved (Figure 3B). One year after surgery, the appearance of foot returned to normal (Figure 3C). A radiograph taken 1 year after surgery also showed that the sequence of the fifth toe column and the width of forefoot had returned to normal (Figure 3D). The fifth digit column sequence was restored (Figure 4A). In Figure 4A, the red dots represented the centers of the lateral metatarsal bones, and the green dots represented the centers of the medial metatarsal bones. The blue line represents the axis of the fifth metatarsal, and the yellow line represents the axis of the fourth metatarsal. In Figure 4B, the blue line represents the axis of the fifth metatarsal bone after surgery, and the blue dot represents the center of the fifth metatarsal bone after surgery; the sequence of the fifth toe line returned to normal after surgery. Postoperatively, the toe line was lengthened, got a natural appearance (Figure 5A). The appearance of left foot recovered well 1-year after operation, and the length of the fifth toe was the same as that of the unimpaired side (Figure 5B). The fifth digit column of left foot was lengthened. the postoperative length of the fifth metatarsal in the left foot was 39 mm and the fifth metatarsal in the right foot was 35 mm. The arch of the foot was completely restored (Figure 6A). The height of the fifth metatarsal bone of left foot was 14.68 mm (Figure 6B). The medial arch of left foot was 114° and the lateral arch was 145° (Figure 6C). The height of the fifth metatarsal of right foot was 14.30 mm (Figure 6D). The medial arch of the right foot was 118° and the lateral arch was 145°. The reconstructed lateral arch was similar to the unimpaired side.

## Discussion

Here, we have described the use of on-top plasty technique to treat postaxial polydactyly between the 4th and 5th metatarsals with an abnormally large forefoot. Many scholars have attempted to apply the existing polydactyly classifications to guide the surgical treatment of polydactyly. However, there is no standard classification (1, 2, 4–7). Based on the metatarsal morphology, Venn-Watson (4) proposed the classification of metatarsal polydactyly into complete duplication, Y metatarsal type, T metatarsal type, enlarged metatarsal type, and soft tissue vegetative type. However, the polydactyly involved in this study could not be classified with this system. Better classifications systems is required.

Uda et al. (5) have discussed in detail the principles and methods of excision procedures for polydactyly. According to their recommendations, decisions about excision should be made based on the appearance of the extra digits. That is, if the appearance of the medial toe was satisfactory, the lateral toe should be removed, and if the outer toe has a satisfactory appearance, the inner toe should be resected. If both toes are similar in terms of appearance, the lateral toe should be removed. With regard to metatarsal polydactyly, the toe column to be removed was mainly assessed based on the imaging findings: if the medial toe column was well-developed, the lateral column should be removed, and if the lateral column was well-developed, the medial column should be removed. In our study, there was no well-developed bone in the medial row (metatarsal dysplasia), but the appearance of the toe was good and the metatarsal joint had a normal axis. Further, the lateral column had a stable metatarsal tarsus and the proximal part of the metatarsal bone was well-developed, but the metatarsophalangeal joint was malformed. According to our previous experience, neither medial nor lateral column resection could have achieved good results. Instead, we opted for the on-top plasty technique, with which we were able to achieve good results.

The on-top plasty technique was widely used in the treatment of repeated bunions and has achieved very good results (8, 9). Based on the promising results, several scholars tried to apply this technique to the treatment of polydactyly. Usami et al. (3) were the first to report the use of the on-top plasty technique to correct short toe deformity in four patients. However, none of their patients had an unusually wide forefoot, and the author performed osteotomy and transposition at the phalangeal level. Their final results showed that the length of the reconstructed toe was increased to different degrees and its appearance was improved. In another study, Han et al. (10) used metatarsal transfer to treat a series of rare types of polydactyly and achieved good results. Since the lateral column malformation in the polydactyly involved in the study occurred at the metatarsal neck level, osteotomy and metastasis at the phalangeal level could not solve the wide anterior foot malformation. Therefore, we used a different approach in which the medial and distal part of the medial row was preserved (which improved the appearance of the good toe and the normal axis of the metatarsophalangeal joint), and the proximal end of the lateral row metatarsophalangeal was “spliced” to form a perfect toe row. With this modified technique, the complete osteoarticular structure and weight-bearing structure of foot were well-reconstructed.

Many scholars are concerned that surgery of the fifth metatarsal will affect weight-bearing activities of the foot. The fifth metatarsal bone is an important part of the transverse arch and plays a key role in walking function (11). In our study, the malformation occurred at the metatarsal neck, so the malformed metatarsal-toe joint in the lateral column needed to be removed in order to correct the forefoot width and relieve pain. During the operation, we not only removed the malformed metatarsal-toe joint in the lateral column, but also reconstructed the distal part of the medial column to ensure that the appearance and bone structure were good. Specifically, the medial column and lateral column were used to reconstruct a complete toe column, and toe lengthening was also performed. The results were very good. For the metatarsal neck deformity, we used metatarsal neck osteotomy and transfer to correct the appearance and reconstruct the arch of the foot so that it was close to the normal physiological state.

To conclude, in the present case of polydactyly, simple excision of the digits did not yield good results. Therefore, we selected the beneficial parts of the medial and lateral columns for combined reconstruction. We performed the first metatarsal osteotomy and transfer at the neck level using the on-top plasty technique to improve appearance and function. We believed that the most important objective when planning a multi-toe operation is to preserve all the beneficial parts for reconstruction. That is, the aim should not be simple toe excision; instead, a tailored, personalized approach was very important. The short-term results in our study were excellent, but long-term follow-up is needed to identify complications that might be missed. Nevertheless, we believe that it is important to preserve all the beneficial parts for reconstruction and to devise a personalized treatment plan.

## Data Availability Statement

The original contributions presented in the study are included in the article/supplementary material, further inquiries can be directed to the corresponding author/s.

## Ethics Statement

The studies involving human participants were reviewed and approved by the Ethics Committee of Children's Hospital of Nanjing Medical University. Written informed consent to participate in this study was provided by the participants' legal guardian/next of kin.

## Author Contributions

WL and LJ wrote the first draft of the manuscript. PZ contributed to conception, design of the study, and revised the manuscript. All authors participated in every revision and improvement of the manuscript and read and approved the final manuscript.

## Conflict of Interest

The authors declare that the research was conducted in the absence of any commercial or financial relationships that could be construed as a potential conflict of interest.

## Publisher's Note

All claims expressed in this article are solely those of the authors and do not necessarily represent those of their affiliated organizations, or those of the publisher, the editors and the reviewers. Any product that may be evaluated in this article, or claim that may be made by its manufacturer, is not guaranteed or endorsed by the publisher.
